# Guide snoRNAs: Drivers or Passengers in Human Disease?

**DOI:** 10.3390/biology8010001

**Published:** 2018-12-20

**Authors:** Manisha Deogharia, Mrinmoyee Majumder

**Affiliations:** 1 Department of Anatomy and Structural Biology, Albert Einstein College of Medicine, Bronx, New York, NY 10461, USA; manisha.deogharia@einstein.yu.edu; 2 Department of Biochemistry and Molecular Biology, College of Medicine, Medical University of South Carolina, Charleston, SC 29425, USA

**Keywords:** snoRNA, RNA modification, box C/D, box H/ACA, 2’-*O*-methylation, pseudouridine, human disease

## Abstract

In every domain of life, RNA-protein interactions play a significant role in co- and post-transcriptional modifications and mRNA translation. RNA performs diverse roles inside the cell, and therefore any aberrancy in their function can cause various diseases. During maturation from its primary transcript, RNA undergoes several functionally important post-transcriptional modifications including pseudouridylation and ribose 2′-*O*-methylation. These modifications play a critical role in the stability of the RNA. In the last few decades, small nucleolar RNAs (snoRNAs) were revealed to be one of the main components to guide these modifications. Due to their active links to the nucleoside modification, deregulation in the snoRNA expressions can cause multiple disorders in humans. Additionally, host genes carrying snoRNA-encoding sequences in their introns also show differential expression in disease. Although few reports support a causal link between snoRNA expression and disease manifestation, this emerging field will have an impact on the way we think about biomarkers or identify novel targets for therapy. This review focuses on the intriguing aspect of snoRNAs that function as a guide in post-transcriptional RNA modification, and regulation of their host genes in human disease.

## 1. Introduction

**Figure 1:**
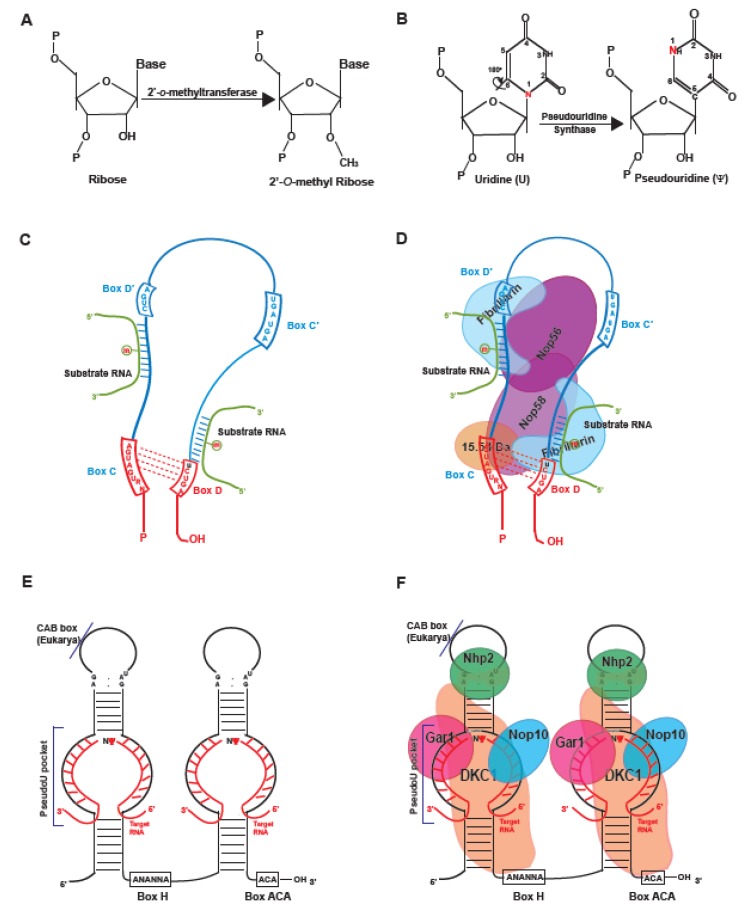
snoRNA guided modifications and machinery. (**A**) Schematic representation of a 2’-*O*-methylated ribose. The 2’-OH group of ribose sugar is *O*-methylated either by a stand-alone methyltransferase or by box C/D guide RNPs. (**B**) Schematic representation of pseudouridine (Ψ). Ψ is a rotational (C-5 ribosyl) isomer of uridine (U), in which the C–C glycosidic bond is broken to form an N–C bond. (**C**) The secondary structure of a box C/D snoRNA. Boxes C, D (red), C’, and D’ (blue) are shown here. “m” represents the target 2’-*O*-methylation site that is always the fifth nucleotide from box D or D’. (**D**) Eukaryal box C/D snoRNP with all the protein components including methyl transferase Fibrillarin (light blue), NOP56 (dark violet), NOP58 (light violet)) and asymmetrically bound 15.5 kDa only at the C/D box (orange). (**E**) Schematic of a eukaryal box H/ACA pseudouridylation guide snoRNA. CAB box is found at the apical loop in some eukaryotic box H/ACA RNAs. The conserved box H (ANANNA) and ACA motifs are located at the 3´ end of the 5’- and 3’- hairpin unit, respectively. Base-pairing of the target RNA (red) with complementary sequences in the pseudouridylation pocket positions the unpaired substrate uridine at the tip of the pocket. The approximately 14-16-nucleotide distance between the modified nucleotide (Ψ) and box ACA (or H) is somewhat maintained. (**F**) Structure of a box H/ACA snoRNP. DKC1 (orange) interacts with box ACA or box H, the pseudouridylation pocket, and NOP10 (sky blue) and GAR1 (pink). As eukaryal box H/ACA RNAs lack kink turn near the apical loop like their archaeal counterpart, NHP2 (green) interacts with the preformed box H/ACA snoRNP in the presence of guide-DKC1-NOP10-GAR1.

Nuclear RNA undergoes numerous nucleoside specific post-transcriptional modifications. Copious evidence now exists to show that critical cellular processes such as maturation of RNA, stabilization of RNA 3D structure, ribosome assembly, protein translation, and recognition steps of tRNAs are dependent on the presence of modified nucleosides on RNA that are conserved from bacteria to humans [1,2,3,4,5,6]. Over 100 chemically distinct modified nucleosides have been identified to date [7]. In RNA, methylation of the ribose moiety at the 2’-hydroxyl group (Figure 1A) and conversion of uridines into pseudouridines (Ψ) (Figure 1B) are the two most prevalent post-transcriptional modifications. These modifications can occur by 1) guide RNA dependent or 2) guide RNA independent (protein only) mechanisms. Eukaryotic small nucleolar RNAs (snoRNAs) are a group of non-coding RNAs that function as the “guide” in the guide RNA dependent RNA modifications. These guide RNAs are required to bind to specific complementary sequences on the substrate RNAs to form a guide-target duplex where a target nucleoside is modified by the RNA binding proteins (RBP), serving as enzymes. These 60–300-nucleotide-long RNAs are primarily located in the nucleolus (snoRNAs) or nucleoplasmic Cajal bodies (small Cajal body-specific RNAs, scaRNAs), a nuclear sub-organelle that is enriched in RNA and RBPs [8]. These snoRNAs can be classified into two major groups based on specific structural features (i) box C/D (Figure 1C) and (ii) box H/ACA (Figure 1E) [9]. To function as a guide, snoRNAs form small nucleolar ribonucleoprotein (snoRNP) complexes with RBPs along with accessory proteins. 2’-*O*-methylation is produced by box C/D snoRNAs which associate with four core proteins: Fibrillarin, NOP56, NOP58, and 15.5 kDa (Snu13p in yeast, L7Ae in archaea) (Figure 1D), where Fibrillarin is the methyltransferase [10]. Box H/ACA snoRNA forms RNP complex with dyskerin (NAP57 in mammals, CBF5 in yeast and archaea), GAR1, NOP10, and NHP2 (L7Ae in archaea) (Figure 1F) to guide Ψ-formation in RNA where dyskerin (DKC1) is the pseudouridine synthase [11,12]. 

snoRNAs are mostly generated from mRNA intronic regions, without a 5’-cap [13], after concomitant splicing, debranching, and trimming [14,15,16]. Additionally, a subset of snoRNAs is also generated from intergenic locations and independent promoters (contain 2,2,7-trimethylguanosine (TMG) cap structure) [17]. Specific non-intronic genes are found to code for important snoRNAs like U3, U8, and U13 that play a role in pre-rRNA endonucleolytic process. Genes harboring snoRNAs in their introns are called host genes [8,18]. There are over 200 different host genes present, out of which some are also non-protein-coding genes with short and poorly conserved open reading frames [14]. These non-protein coding host genes were thought to have no function other than to carry the snoRNA-encoding sequences in their introns [19]. However, recent data suggest a functional role for these host genes. For example, a mouse noncoding RNA Zfas1 gene intronically hosts three box C/D snoRNA [20]. Zfas1 is down-regulated in ductal carcinoma tumor tissues. Knockdown of this RNA in mouse carcinoma cell line shows an increase in different cell proliferation and differentiation markers, although the levels of snoRNAs generated from this RNA remain relatively constant [20]. 

The protein-coding host genes are involved in nucleolar function, ribosome structure formation, and protein synthesis [21]. The host genes are also of particular importance and physiologically pertinent as their intronic snoRNAs eventually participate in ribosome biogenesis [8,21]. Interestingly, in humans, many of the host genes belong to a family of terminal oligopyrimidine (TOP) genes [22]. This set of genes codes for proteins involved [19] in transcription, translation, and growth-dependent regulation [17,22]. 

In recent years, independent studies using various approaches have concluded that apart from their housekeeping role, snoRNAs also control cell fate and oncogenesis [23,24,25,26,27]. There are very informative reviews available discussing the different roles of snoRNAs or RNA modifications, and disease [8,28,29]. However, the primary scope of this review is to unite the guide RNAs and their host genes, with abnormalities in expression that are primarily or secondarily responsible for human diseases. 

## 2. Box H/ACA Guide RNAs in Human Disease

Pseudouridine (Ψ) was discovered in 1951 as the most abundant post-transcriptionally modified nucleotide, and in 1959 it was chemically characterized [30]. Later in the ’90s, different laboratories pioneered in identifying the small nucleolar RNAs that guided the isomerization of uridine into Ψ [31,32]. This subclass of snoRNAs consists of specific structural features called box H/ACA (box H, a variant of box ACA, ANANNA and box ACA, ACANNA; N: any nucleotide) in a double hairpin (60–75 long each) connected by a hinge region (Figure 1E). The distance between the target uridine and the box H or box ACA of the guide RNA is around 14 nt. Additionally, box H/ACA scaRNAs in eukaryotes carry a Cajal body localization signal called CAB box (consensus of ugAG) in the terminal loop of either their 5′- or 3′-hairpins (Figure 1E) [33]. RNA guided pseudouridines are found on various types of RNAs, majorly rRNAs along with spliceosomal snRNAs [34,35,36]. Interestingly, recently characterized nucleoplasmic AluACA RNAs with unknown function, derived from intronic Alu elements, show a similarity in structure to that of box H/ACA scaRNAs [37]. 

Large subunit (LSU: 28S, 5.8s, and 5S) and small subunit (SSU: 18S) rRNAs are the two major RNA components of the ribosome where around 100 uridines are converted to pseudouridines by box H/ACA snoRNPs [33,38]. For example, the RNA encoded by snoRA12 guides Ψ formation at position 372 on 28S rRNA and snoRA24 (ACA24) modifies positions 609 and 863 on 18S rRNA. Moreover, the two most conserved Ψs on 28S rRNA are modified by snoRA74 (U19) at least in the higher eukaryotes (also see Section 2.1). Ψs are known to provide structural stability to the modified RNAs [39] that are required for the biogenesis of ribosome [40]. In the sections below, we describe the box H/ACA guide RNAs and their host genes that are connected to numerous diseases due to their aberrant expression. We have extensively used databases and literature [15,16,41,42,43] to check for all the predicted and verified modifications to date.

### 2.1. Box H/ACA Guide RNAs in Cancer

Studies show that pseudouridines can be found at functionally critical positions on the rRNAs [3,38,42,43]. Thus, it can be projected that deregulation of box H/ACA snoRNAs that guide Ψ formation can be involved in cancer [44]. For example, one of the recently identified snoRNAs (h5sn2) with box H/ACA features shows a drastic reduction in expression in meningioma compared to the healthy brain [45]. Meningioma, the most common kind of slow-growing brain tumor, is found in the meninges, the membranes that edge the brain and spinal cord. snoRNA h5sn2 shows a sequence complementarity to human 5S rRNA. 

While the loss of h5sn2 shows a potential tumorigenic activity, snoRA42 is overexpressed in non-small cell lung cancer (NSCLC), one of the primary causes of cancer deaths across the world [46]. snoRA42 is highly expressed in colorectal cancer as well [47]. This study also shows that over-expression of snoRA42 induces cell proliferation, migration, invasion, and tumorigenicity in colorectal cancer [47]. snoRA21 shows oncogenic properties in human colorectal cancer as well [48]. 

Expression of two other snoRNAs, snoRA47 and ACA11, are also high in hepatocellular carcinoma tissues compared to normal tissues [49,50]. snoRA74 (U19), responsible for modifying the two most conserved Ψs on 28S rRNA, 3741 and 3743 is found to be up-regulated in gallbladder cancer [51]. These positions are conserved from bacteria (protein only modification) to humans [1,38,41]. Both Ψs are located at a central region of the ribosome called the decoding center which is responsible for contacting the passing tRNAs [41]. Irregular expression of a group of box H/ACA snoRNAs has also been shown in hematological disorders like acute myeloblastic and lymphoblastic leukemia, T-cell lymphoma and multiple myeloma [52,53,54,55]. 

To date, there is no direct evidence of either deregulation of a particular snoRNA guided modification or mere presence or absence of modification as a sole cause of any disease. However, in head and neck squamous cell carcinoma (HNSCC) a different form of snoRA71C is found with an A60>G60 nucleotide mutation in the box H region. This substitution seems to alter the guide-target base pairing that is required to modify position 406 on 18S rRNA [29,56]. We can speculate that this A to G alteration followed by a lack of modification can indeed be the cause or a trigger in HNSCC initiation or progression.

Additionally, box H/ACA scaRNAs that modify spliceosomal RNAs are also associated with cancer when deregulated. For example, scaRNA3, also known as HBI-100, is found to be up-regulated in breast cancer [57]. Several other box H/ACA guide RNAs, associated with cancer are listed in Table 1 and Table 2.

### 2.2. Box H/ACA Guide RNAs in Genetic Disease

Apart from cancer, box H/ACA RNAs are also associated with genetically inherited diseases when altered. A subset of snoRNAs is down-regulated in a bone marrow failure syndrome called X-linked dyskeratosis congenita (X-DC) [29,52,53,54,55,58]. DC (X-DC, and two other subtypes of DC-autosomal dominant or autosomal recessive), is a congenital disorder with defects including bone marrow failure, skin abnormalities, hematopoietic malignancies, and pulmonary fibrosis [52,59]. A study by Bellodi et al. shows that some box H/ACA snoRNAs like snoRA15, snoRA24, snoRA31, snoRA48, snoRA56, and snoRA67 are down-regulated in fibroblasts or lymphoblast cells expressing mutant *DKC1* as found in X-DC patients [29,52]. scaRNA U93 is down-regulated in the majority of the X-DC patients’ cells tested except CD34+ hematopoietic progenitor cells with *DKC1* promoter mutation at position C-141 to G (c.-141, C>G) [52]. However, snoRA42 is found to be significantly up-regulated in lymphocytes expressing *DKC1* with N-terminal L37 deletion (ΔL37). Interestingly, these H/ACA snoRNA guided modifications are found mostly within two distinct regions of the ribosome which include domain II of 28S rRNA and expansion segment 6 (ES6) on 18S rRNA [60]. The same study by Bellodi et al. also shows a reduction in Ψ formation at 109, 119, 572, 1367, and 1445 residues on 18S rRNA by using liquid chromatography-tandem mass spectrometry (LC-MS/MS) [52]. The reduction in Ψs is found in fibroblasts with *DKC1* (ΔL37) or lymphoblasts with *DKC1* mutation at position T66 to A (T66A) where a decrease in their respective guide RNAs is also observed except for snoRA42. 

Box H/ACA guide RNAs are also involved in a genetic disorder called tetralogy of Fallot (TOF or “TET”) in infants. TOF is a condition of numerous related congenital heart defects that are present at birth. It occurs due to abnormal development of the fetal heart during the first eight weeks of pregnancy. Three box H/ACA scaRNAs, scaRNA1, scaRNA4, and scaRNA8, have been found to be down-regulated in TOF in a recent study by Nagasawa et al. [61]. These scaRNAs are responsible for U2 snRNA modification.

### 2.3. Box H/ACA Guide RNA Host Gene Deregulation in Disease

Evidence to date suggests that apart from a handful of snoRNAs, many of the altered box H/ACA snoRNA expression in different disorders is independent of the host gene transcription [53,54]. The evidence supports either of two hypotheses: box H/ACA snoRNA deregulation can directly associate with a disease devoid of the host gene modulation, or the regulation between host genes and their intronic snoRNAs is yet to be identified. However, there are ample examples of host gene deletion or mutation causing disease of which some cases may show an alteration in their intronic box H/ACA RNAs [27]. For example, two box H/ACA-type snoRNA genes, snoRA6 and snoRA62 are encoded from the host gene RPSA or Laminin receptor (LAMR). These RNAs modify positions 3616 and 3830 on 28S rRNA [38]. There are mutations found in the *LAMR/RPSA* gene which can be linked to congenital asplenia, a dysfunction of the spleen [62]. Future studies will be needed to show if *RPSA* mutations impact these snoRNA expressions.

Another ribosomal protein-coding gene *RPL5*, where over 15 different mutations have been identified so far, is associated with Diamond-Blackfan anemia (DBA), an inherited bone marrow failure syndrome [63]. *RPL5*-mutation induced pluripotent stem cells (iPSCs) from DBA patients exhibit defective 60S ribosomal subunit assembly, accumulation of 12S pre-rRNA, and impaired erythropoiesis [64]. *RPL5* hosts snoRA66 which guides Ψ119 on 18S rRNA. Not much work has been done to show if there is a correlation between *RPL5* mutation and snoRA66 expression followed by Ψ119 formation. Interestingly, as mentioned in Section 2.2, X-DC patients’ cells show a decrease in Ψ119 formations in lymphocytes and fibroblasts expressing *DKC1* (T66A) and *DKC1*, respectively [52]. Additionally, snoRA66 is also down-regulated in both cells expressing mutant *DKC1*. Like X-DC, it would be intriguing to see if snoRA66 expression and Ψ119 formation are also regulated along with *RPL5* in DBA.

Additionally, *DKC1*, where mutations are found in almost all X-DC patients, also acts as the host gene for snoRA36 and snoRA56 [59,65]. *DKC1* mutations not only affect its protein expression and function in DC patients, but they may also affect the expression of these two snoRNAs. snoRA36 and snoRA56 modify positions 105 and 1244 on 18S and 1664 on 28S and 296 on 18S rRNA, respectively [52,59,65]. DKC1 is often down-regulated in DC along with certain snoRNAs (see Section 2.2) [52,59,65]. Though reports exist suggesting that *DKC1* exerts its function independent of the snoRNAs that it hosts, further investigation is necessary to test this link. It is possible that both host genes and their intronic snoRNAs may be relevant under specific cellular conditions.

We summarize the box H/ACA snoRNAs and scaRNAs with validated or predicted targets and the snoRNA host genes that are associated with human disease in Table 1 and Table 2. 

## 3. Box C/D Guide RNAs in Human Disease

Another post-transcriptional modification, guided by snoRNAs, is the 2’-*O*-methylation of the ribose sugar. This modification is brought about by box C/D snoRNAs. Box C/D snoRNAs in eukaryotes are characterized by the presence of a consensus box C (RUGAUGA) and box D (CUGA) near the 5’ and 3’ ends of the RNA, respectively (Figure 1C). They also contain imperfect copies of these two boxes, box C’ and box D’ in between the two C and D boxes. The region between the boxes (C and D’) and (C’ and D) form base pairing with target RNAs where the snoRNP methylates the 5th nucleotide upstream of D or D’ boxes. Like box H/ACA scaRNAs, box C/D scaRNAs are also found in Cajal Bodies. They do not have a CAB box like box H/ACA scaRNAs, but they contain G.U/U.G wobble stem instead, which helps their translocation to Cajal bodies [78]. Methylation of the 2’- hydroxyl group favors the C3’-endo base conformation in both purines and pyrimidines which enhances base stacking and makes the RNA more rigid. 2’-*O*-methylation has been proposed to stabilize the RNA and protect it from the attack of ribonucleases at higher temperatures [79].

In humans, the mapping of 2’-*O*-methylation in rRNA reveals most modifications to be present in comparable amounts for different cell lines. However, few specific sites show a different degree of modifications between HeLa, cervical cancer and HCT116, colon cancer cell lines [42]. This observation could mean that 2’-*O*-methylation in different cancers modify the functions of ribosomes to different extents. Lack of 2’-*O*-methylations has not been directly linked to any disease, however; box C/D snoRNAs have been implicated in different diseases. 

### 3.1. Box C/D Guide RNAs in Cancer

The expression of a bulk of box C/D snoRNAs is altered in different cancers. SNORD50 guides methylation at C2848 and G2863 on 28S rRNA and has been designated as a tumor suppressor gene for prostate, colon, and breast cancers [23,24,80]. Deletion of two base pairs in the locus of this snoRNA has been associated with prostate cancer [24]. Ectopic expression of this snoRNA gene is shown to reduce the tumorigenicity of prostate cancer cells that are marked by a reduced colony forming ability of two cell lines 22Rv1 and LNCaP [24]. While screening for genes that are sensitive to metabolic stress in diabetes and other metabolic syndromes, an independent study identified SNORD32a, SNORD33, and SNORD35a in Chinese Hamster ovary (CHO) cells and C2C12 mouse myoblasts [81]. Suppression of the three snoRNAs expression renders C2C12 myoblasts resistant to Palmitate-induced and general apoptosis (also see Section 3.3) [81]. Although these experiments are carried out in different cell types, the finding that these snoRNAs are involved in response to general oxidative stress can also be applicable during oncogenesis. However, further investigation is needed to support this link. Increased and decreased expression of various snoRNAs and scaRNAs with known targets in 18S rRNA, 28S rRNAs, and snRNAs, respectively have been linked to several types of leukemia, prostate cancer and multiple myeloma (reviewed in reference [44]) and also listed in Table 3 and Table 4.

### 3.2. Box C/D Guide RNAs in Genetic Disease

The well-characterized study done on box C/D snoRNA is for Prader Willi syndrome (PWS) [82,83]. Mental retardation, muscle hypotonia, obesity, and shorter height are the disease phenotypes. Deregulation of two box C/D snoRNAs, SNORD115 and SNORD116 have been directly linked to this disease. SNORD115 and SNORD116 originate from chromosomal locus 15q11-q13, a region that contains several genes controlled by genomic imprinting. It is a phenomenon in which genes are expressed preferentially from one parental origin. Interestingly, these snoRNAs are remarkably modulated in PWS only when there is an inheritance from paternal 15q11-q13 deletion; however, their expression seems unchanged in the much less severe Angelman syndrome when inherited as a maternal 15q11-q13 deletion [84]. Both SNORD115 and SNORD116 are bona fide box C/D snoRNAs [82] which do not undergo hydrolysis to form smaller snoRNA products in either human or mouse brains, but their targets have not yet been found, and thus it is beyond the focus of this review for detailed discussion. 

Recently, it has also been shown that 12 sno/scaRNAs with known methylation targets in U2 and U6 snRNAs are down-regulated in congenital heart defects [58]. As described in Section 2.2, along with the H/ACA scaRNAs several box C/D snoRNAs and scaRNAs are down-regulated in TOF [61]. These box C/D scaRNAs are guides for modifications on U2 snRNA (Table 4). Some of them are also predicted to guide rRNA modification but are not yet validated. 

### 3.3. Box C/D Guide RNAs in Other Disease

As mentioned in Section 3.1, snoRNAs have also been associated with lipotoxicity, a metabolic stress associated with diabetes and obesity [81]. Increased levels of three box C/D snoRNAs, SNORD32a, SNORD33, and SNORD35a, were seen in CHO cells when they were exposed to fatty acids. snoRNAs were a surprise element in this study, which was carried out as a genetic screen in CHO cells using retroviral promoter trap mutagenesis to create single gene disruptions to select for cells, which could grow in a lipotoxic environment. One of the mutant cell lines, which grew in this environment, had a disruption in the locus for ribosomal protein L13a (*RPL13a*) [81]. There was no change in rRNA methylation in cells under lipotoxic stress although 12-13 bp in each of the three snoRNAs matched with potential sites in 18S and 28S rRNA. Interestingly, unlike their usual locations in the nucleolus, these snoRNAs accumulated in the cytoplasm in C2C12 mouse myoblasts under lipotoxic conditions [81]. Knockdown of the three snoRNAs also reduced the amount of H_2_O_2_ derived reactive oxygen species in these cells and helped the cells evade apoptosis. These snoRNAs are also key players in an in vivo oxidation stress as mouse model studies show with lipopolysaccharide administration as compared with saline-treated mouse controls showed increased levels of these snoRNAs in liver [81]. 

In another case, patients with anterior cruciate ligament (ACL) injury show an increased level of snoRNAs SNORD38 and SNORD48 in their serum as compared with controls [85]. However, it is not clear if these are secreted in serum or released by damaged cells. Similar circulating snoRNAs, as well as other RNAs, are detected in serum in pancreatic cancers (reviewed in [86]). SNORD38 level is up-regulated in all ACL patients. However, the SNORD48 level is even higher in ACL patients with cartilage damage than the normal donors or patients with ACL but no cartilage damage. Thus, snoRNAs can also be used as a biomarker for early diagnosis of the cartilage damage associated with this disease [85].

### 3.4. Box C/D Guide RNA Host Gene Deregulation in Disease

Like box H/ACA RNA host genes, there are several genes that produce box C/D RNAs from their intronic regions and also not much work has been done to link host genes to their snoRNA regulation. Moreover, ribosomal protein genes very commonly serve as host genes for box C/D RNAs as well. *RPS11* encodes SNORD35B that guides C4506 methylation on 28S rRNA. RPS11 protein is up-regulated in colorectal carcinoma [87] and down-regulated in staurosporine-induced apoptotic MCF7 breast cancer cell line [88]. This aberrant expression did not affect ribosome biogenesis in MCF7 cells pointing towards the fact that instead of the protein, the snoRNA encoded from its intron is playing a role in cancer. Recently, SNORD35B has also been found to be up-regulated in HNSCC [89]. *RPL13A* is another host gene encoding SNORD32, SNORD33A, and SNORD35A and deregulation of the host gene or snoRNAs are associated with different types of cancer [26,54] (also see Section 3.1). There is an array of ribosomal protein genes whose introns code for snoRNAs (Table 3 and Table 4) that can cause disease. Additionally, the majority of these snoRNA encoding host genes are targets of Myc transcription factor in Drosophila [14] which is frequently up-regulated in different cancers. 

GAS5 (Growth arrest-specific transcript 5) transcripts regulate both cell death and proliferation and are found to be reduced in both breast cancer and HNSCC compared to normal [25,90]. Additionally, SNORD44, which is one of the ten box C/D snoRNAs that are encoded in *GAS5* introns, is also significantly associated with HNSCC prognosis [25]. 

Here, we have summarized the known box C/D snoRNAs and scaRNAs which have been observed to play a role in numerous diseases with their corresponding host genes and verified or predicted targets in Table 3 and Table 4.

## 4. Conclusions

snoRNAs have been known for decades, mostly for their role in ribosomal biogenesis and modification. Recent developments in sequencing techniques have helped to detect snoRNAs and their further processed products. Many snoRNAs are processed into shorter functional forms like miRNA [99], but this process is still not fully understood. A recent study shows that human noncoding microRNA-1291 (hsa-miR-1291) is localized within the box H/ACA RNA snoRA34, and targets multidrug resistance-associated protein 1 (MRP1/ABCC1) mRNA 3′-untranslated region (3′UTR) [100]. 

The contribution of the non-coding RNAs to human disease is a relatively new area of research, predominantly based on the recent findings that their expression and function are often altered in diseases such as cancer, genetic, and neurological disorders [28,29]. So far, the mechanisms for to how snoRNA or snoRNA-derived molecules carry out their varied regulatory roles are not very clear and thus are an area for future research. Few questions that are critical to understanding the link between snoRNAs and host genes are: why are different snoRNAs that are encoded from the same host gene differentially altered in diseases? For example, SNORD32, SNORD33A, and SNORD35A although expressed from the same host gene *RPL13A* are differentially modulated in different carcinomas (see Table 3). Another host gene, *SNHG1* encodes eight snoRNAs of which only a handful are up-regulated in multiple myeloma (see Table 3). It will be interesting to see if there is a finer regulation on which certain snoRNAs are up-regulated, or they are all up-regulated, and some can escape detection merely based on their half-lives. Additionally, a potential feedback loop, present between the host genes and their intronic snoRNAs, may also regulate either of their expressions under specific diseased states.

Still, there are many snoRNAs, in higher eukaryotes whose targets and functions are not yet known. These are called orphan snoRNAs. Target sequences for the orphan guides can possibly be present within mRNAs. Alteration in the guide RNA expression can trigger changes in the mRNA modification landscape and, consequently can alter protein translation. There are many open-ended questions like these, and the answers will require a combination of bioinformatics, biophysical and biochemical experimental approaches to provide understanding into the detailed macromolecular modulations that are induced by post-transcriptional nucleoside modifications. This will undoubtedly open up new avenues of research for better disease prognosis and therapeutic interventions.

## Figures and Tables

**Table 1:** Box H/ACA guide snoRNAs in human disease.

Guide RNA	Target rRNA Position (Uridine)	Host Gene	Disease Associated with the RNA	Level Compared to Normal	Ref.
snoRA2A (ACA2A)	28S-4263, -4282	*KANSL2* (KAT8 regulatory NSL complex subunit 2)	Breast cancer	Up	[66]
snoRA6 (ACA6); snoRA62 (E2)	28S-3616, -4438, and -3830, -3832	*RPSA* (ribosomal protein SA, 67 kD laminin receptor)	Isolated congenital asplenia	Down-regulation of RPSA due to mutations	[62]
snoRA15 (ACA15)	18S-1367	*CCT6A* (chaperonin containing TCP1, subunit 6A (zeta 1))	Acute myeloblastic, acute lymphoblastic and peripheral T-cell lymphoma, X-Linked DC	Down	[29,52]
snoRA21 (ACA21)	28S-4401, 18S-1499	*RPL23* (ribosomal protein L23)	Colorectal cancer	Up	[48]
snoRA22 (ACA22)	28S-4966 (snoRA33 *), -4975	*CCT6P1* (chaperonin containing TCP1 subunit 6 pseudogene 1)	DC	Down	[65]
snoRA23 (ACA23)	28S-3737, -4331	*IPO7* (importin 7)	Pancreatic Cancer, DC	Up	[43,67]
snoRA24 (ACA24)	18S-609, -863, 18S-1045 (snoRA24B *)	*SNHG8* (Small Nucleolar RNA Host Gene 8)	Acute myeloblastic, acute and chronic lymphoblastic, and peripheral T-cell lymphoma, X-Linked DC	Down	[29,52]
snoRA27 (ACA27)	28S-3694, -4522	*RPL21* (ribosomal protein L21)	HHS	-	[68]
snoRA31 (ACA31)	28S-3713, 18S-218	*TPT1* (Tumor protein, translationally-controlled 1)	Musculoskeletal aging and osteoarthritis	Up	[69]
snoRA36 (ACA36)	18S-105, -1244	*DKC1* (dyskeratosis congenita 1, dyskerin)	DC, X-Linked DC	Down	[65]
snoRA40 (ACA40)	28S-4546, 18S-1174	*TAF1D* (TATA-box binding protein-associated factor, RNA pol I subunit D)	Multiple myeloma of the TC1 subgroup, Asthma, Multiple sclerosis	Up	[54,70,71]
snoRA42 (ACA42)	18S-109, -572 (snoRA80 *)	*KHDC4* (KH Domain Containing 4)	NSCLC, X-Linked DC	Up	[46,72]
snoRA44 (ACA44)	18S-686, -822, -897	*SNHG12* (small nucleolar RNA host gene 12)	Hepatocellular carcinoma	Host gene up-regulation	[73]
snoRA47 (HBI-115)	28S-1766	*ZBED3* ( zinc finger BED-type containing 3)	Hepatocellular carcinoma	Up	[49]
snoRA48 (ACA48)	28S-3797	*SENP3-EIF4A1* (eukaryotic translation initiation factor 4A1)	Breast Cancer	Down	[29]
snoRA56 (ACA56)	28S-1664, 18S-296	*DKC1*	DC, X-Linked DC	Down	[52,59]
snoRA64 (U64)	28S-4975	*RPS2* (ribosomal protein S2)	Multiple myeloma, Prostate cancer, X-DC	Up	[29,52,54]
snoRA66 (U66)	18S-119	*RPL5* (ribosomal protein L5)	Diamond-Blackfan Anemia	Up	[63]
snoRA67 (U67)	18S-1445	*SENP3-EIF4A1*	X-Linked DC	Down	[52]
snoRA71C, D (U71C, U71D)	18S-406	*SNHG17* (small nucleolar RNA host gene 17)	Myelofibrosis, a variant is expressed in HNSCC	Down	[53,56]
snoRA74A (U19)	28S-3741, -3743 and U3-8	*MATR3* (Matrin 3)	Astrocytoma, Gallbladder cancer	Up	[51,74]
snoRA81 (HBI-61)	28S-4606	*EIF4A2* (eukaryotic translation initiation factor 4A, isoform 2)	DC	Down	[75]

* also modifies this position, ( ) alternative names used in the literature.

**Table 2:** Box H/ACA guide scaRNAs in human disease.

Guide RNA	Target snRNA Position (Uridine)	Host Gene	Disease Associated with the RNA	Level Compared to Normal	Ref.
scaRNA1 (ACA35)	U2-89	*PPP1R8* (protein phosphatase 1 regulatory subunit 8)	Tetralogy of Fallot (TOF), a heart condition in children	Down	[61]
scaRNA4 (ACA26)	U2-41	*KHDC4* (KH domain containing 4, pre-mRNA splicing factor)	TOF	Down	[61,76]
scaRNA8 (U92)	U2-34, -43, -44	*HAUS6* (HAUS augmin like complex subunit 6)	TOF	Down	[61,76]
scaRNA11 (ACA57)	U5-43	*CHD4* (Chromodomain helicase DNA binding protein 4)	Sifrim-Hitz-Weiss syndrome	Host gene mutation	[77]
scaRNA13 (U93)	U2-54, U5-51	*SNHG170* (small nucleolar RNA host gene 170)	DC, Congenital heart defects	Down	[58]
scaRNA23 (ACA12)	U6-40	*COP1* (COP1, E3 ubiquitin ligase)	Breast Cancer	UP	[57]

( ) alternative names used in the literature.

**Table 3:** Box C/D guide snoRNAs in human disease.

Guide RNA	Target rRNA/snRNA Position	Host Gene	Disease Associated with the RNA	Level Compared to Normal	Ref.
SNORD7 (Z30)	U6-A47	*LINC02001* (long intergenic non-protein coding RNA 2001)	Tetralogy of Fallot (TOF), a heart condition in children	Down	[61]
SNORD8 (mgU6-53)	U6-A53	*CHD8* (chromo-domain helicase DNA binding protein 8)	TOF	Down	[61]
SNORD9 (mgU6-53B)	U6-A53	*CHD8*	TOF	Down	[61]
SNORD19 (HBII-108)	18S-G683 (SNORD136) *	*GNL3* (G protein nucleolar 3)	Colorectal cancer	Up	[91]
SNORD25 (U25)	18S-G1490	*SNHG1* (small nucleolar RNA host gene 1)	Multiple Myeloma	Up	[92]
SNORD27 (U27)	18S-A27	*SNHG1*	Multiple Myeloma	Up	[92]
SNORD28 (U28)	18S-C1391	*SNHG1*	Breast tumors	Up	[93]
SNORD30 (U30)	28S-A3804 18S-C1383	*SNHG1*	Multiple Myeloma	Up	[92]
SNORD31 (U31)	28S-G4166	*SNHG1*	Multiple Myeloma	Up	[92]
SNORD32 (U32)	28S-A1511 18S-G1328	*RPL13* (ribosomal protein L13)	Secondary plasma cell leukemia	Down	[54]
SNORD33A (U33A)	18S-U1326	*RPL13*	NSCLC	Up	[26]
SNORD35A (U35A)	28S-C4506	*RPL13*	Colorectal carcinomas, Head and neck cancer	Up	[87,89]
SNORD35B (U35B)	28S-C4506	*RPS11* (ribosomal protein S11)	Colorectal carcinomas, HNSCC	Up	[87,89]
SNORD38A (U38A) SNORD38B (U38B)	28S-A1858	*RPS8* (ribosomal protein S8)	ACL	Up	[85]
SNORD43 (U43)	18S-C1703	*RPL3* (ribosomal protein L3)	Breast cancer and HNSCC	Patient-specific variability	[25]
SNORD44 (U44)	18S-A166	*GAS5* (Growth arrest-specific transcript 5)	Breast cancer and HNSCC	Down	[25]
SNORD48 (U48)	28S-C2279	*KIF24* (Kinesin Family Member 24)	ACL	Up	[85]
SNORD50A (U50)	28S-C2848, 28S-G2863	*SNHG5* (small nucleolar RNA host gene 5)	Breast cancer, prostate cancer, colon cancer	Down	[23,24,80]
SNORD66 (HBII-142)	18S-C1272	*EIF4G1* (Eukaryotic translation initiation factor 4 gamma, 1)	NSCLC	Up	[26]
SNORD67 (HBII-166)	U6-C60	*CKAP5* (cytoskeleton associated protein 5)	TOF	Down	[61]
SNORD71 (HBII-239)	5.8S-U14	*AP1G1* (adaptor-related protein complex 1 subunit gamma 1)	T cell lymphoma	Down	[55]
SNORD76 (U76)	28S-A2350	*GAS5*	Hepatocellular carcinoma, glioblastoma and NSCLC	Up in NSCLC	[94,95]
SNORD78 (U78)	28S-G4593	*GAS5*	Hepatocellular carcinoma	Up	[96]
SNORD94 (U94)	U6-C62	*PTCD3* (pentatricopeptide repeat domain 3)	TOF	Down	[61]
SNORD98 (HBII-419)	18S-G867	*CCAR1* (cell division cycle and apoptosis regulator 1)	Colorectal cancer	Up	[91]
SNORD105B	18S-U799	*PPAN* (peter pan homolog)	Gastric cancer	Up	[97]

* also modifies this position, ( ) alternative names used in the literature.

**Table 4:** Box C/D guide scaRNAs in human disease.

Guide RNA	Target snRNA Position	Host Gene	Disease Associated with the RNA	Level Compared to Normal	Ref.
scaRNA2 (HBII-382)	U2-G25 and G61	Independent transcriptional unit	Congenital heart defect and TOF	Down	[58,61]
scaRNA9 (Z32)	U2-G19, A30	Centrosomal Protein 295	Chronic lymphocytic leukemia and TOF	Down	[54,61]
scaRNA17 (U91)	U4-C8 and U12-G22	*SNHG22* (small nucleolar RNA host gene 22)	Chronic lymphocytic leukemia	Down	[54,98]

( ) alternative names used in the literature.
